# Evaluation of a brief virtual implementation science training program: the Penn Implementation Science Institute

**DOI:** 10.1186/s43058-023-00512-5

**Published:** 2023-11-06

**Authors:** Amelia E. Van Pelt, Christopher P. Bonafide, Katharine A. Rendle, Courtney Wolk, Judy A. Shea, Amanda Bettencourt, Rinad S. Beidas, Meghan B. Lane-Fall

**Affiliations:** 1 Department of Medical Social Sciences, Northwestern University Feinberg School of Medicine, 625 N Michigan Ave Suite 2100, Chicago, IL 60611 USA; 2 Penn Implementation Science Center at the Leonard Davis Institute for Health Economics, University of Pennsylvania, 3641 Locust Walk, Philadelphia, PA 19104 USA; 3 Section of Hospital Medicine, Children’s Hospital of Philadelphia, 3401 Civic Center Boulevard, Philadelphia, PA 19104 USA; 4 Department of Family Medicine and Community Health, University of Pennsylvania Perelman School of Medicine, 51 N 39th Street floor 7, Philadelphia, PA 19104 USA; 5 Department of Psychiatry, Perelman School of Medicine, University of Pennsylvania, 3553 Market Street, 3Rd Floor, Philadelphia, PA 19104 USA; 6 Department of Medicine, Division of General Internal Medicine, University of Pennsylvania Perelman School of Medicine, 423 Guardian Drive, Philadelphia, PA 19104 USA; 7 Department of Family and Community Health, University of Pennsylvania School of Nursing, Philadelphia, PA 19104 USA; 8 Department of Anesthesiology and Critical Care, University of Pennsylvania Perelman School of Medicine, 3400 Spruce Street Suite 680, Philadelphia, PA 19104 USA

**Keywords:** Capacity building, Training, Implementation science, Evaluation, Education

## Abstract

**Background:**

To meet the growing demand for implementation science expertise, building capacity is a priority. Various training opportunities have emerged to meet this need. To ensure rigor and achievement of specific implementation science competencies, it is critical to systematically evaluate training programs.

**Methods:**

The Penn Implementation Science Institute (PennISI) offers 4 days (20 h) of virtual synchronous training on foundational and advanced topics in implementation science. Through a pre-post design, this study evaluated the sixth PennISI, delivered in 2022. Surveys measures included 43 implementation science training evaluation competencies grouped into four thematic domains (e.g., items related to implementation science study design grouped into the “design, background, and rationale” competency category), course-specific evaluation criteria, and open-ended questions to evaluate change in knowledge and suggestions for improving future institutes. Mean composite scores were created for each of the competency themes. Descriptive statistics and thematic analysis were completed.

**Results:**

One hundred four (95.41% response rate) and 55 (50.46% response rate) participants completed the pre-survey and post-survey, respectively. Participants included a diverse cohort of individuals primarily affiliated with US-based academic institutions and self-reported as having novice or beginner-level knowledge of implementation science at baseline (81.73%). In the pre-survey, all mean composite scores for implementation science competencies were below one (i.e., beginner-level). Participants reported high value from the PennISI across standard course evaluation criteria (e.g., mean score of 3.77/4.00 for overall quality of course). Scores for all competency domains increased to a score between beginner-level and intermediate-level following training. In both the pre-survey and post-survey, competencies related to “definition, background, and rationale” had the highest mean composite score, whereas competencies related to “design and analysis” received the lowest score. Qualitative themes offered impressions of the PennISI, didactic content, PennISI structure, and suggestions for improvement. Prior experience with or knowledge of implementation science influenced many themes.

**Conclusions:**

This evaluation highlights the strengths of an established implementation science institute, which can serve as a model for brief, virtual training programs. Findings provide insight for improving future program efforts to meet the needs of the heterogenous implementation science community (e.g., different disciplines and levels of implementation science knowledge). This study contributes to ensuring rigorous implementation science capacity building through the evaluation of programs.

**Supplementary Information:**

The online version contains supplementary material available at 10.1186/s43058-023-00512-5.

Contributions to the literature

- This paper describes one approach to meeting the increasing demand for implementation science expertise: building capacity rapidly by training heterogenous groups of learners (e.g., different disciplines and levels of implementation science knowledge) via a brief, virtual institute convened by an implementation science center within a large university.
- This study includes a rigorous evaluation of the sixth iteration of a virtual institute focused on advancing implementation science competencies.
- Findings highlight the strengths and weaknesses of the institute and provide insight into the type of learners seeking out brief implementation science training, which can inform modifications for future capacity building initiatives.

## Background

Implementation science offers an opportunity to systematically close the research-to-practice gap — perhaps one of the greatest current challenges of public health and clinical practice. Over the past two decades, the field has evolved to include a comprehensive repository of approaches that has enabled the advancement of the equitable adoption of evidence-based practices [1–3]. Recognizing the value of implementation science, the number of funding mechanisms (e.g., NIH PAR-22–105), targeted journals (e.g., *Implementation Science*, *Implementation Science Communications*, I*mplementation Research and Practice*, and *Global Implementation Research and Applications*), and academic conferences (e.g., AcademyHealth/NIH Conference on the Science of Dissemination and Implementation in Health) have increased in recent years. To continue this momentum and guarantee the future growth of the field, the development of a robust bench of implementation scientists and practitioners is a priority.

Fortunately, multiple implementation science training programs characterized by different objectives and designs have emerged. A review conducted in 2022 identified 74 capacity building initiatives for dissemination and implementation science [4]. Programs vary in format (e.g., virtual and in-person), duration (e.g., 2-year program and brief 3-day institute), target audience (e.g., funded researchers and students), cost (open access and tuition-based programs), and sponsor (e.g., academic institutions and National Institutes of Health) [4–6]. Such initiatives have contributed to the growth of the field through the development of specific competencies critical for conducting rigorous implementation science. Despite the diverse set of educational initiatives, however, the demand for training in implementation science has outpaced the supply [7, 8].

Current capacity building initiatives may not meet the needs of all learners. For example, the level of interest and application in implementation science include individuals with an awareness of the field but without plans to lead implementation science projects. Other learners include individuals with an understanding of implementation science who seek to incorporate relevant concepts into their projects (i.e., they require foundational training) and individuals with expertise in implementation science who seek to advance the discipline through their work (i.e., “implementation scientists” requiring advanced training) [9]. The training needs and competencies for these different phenotypes will differ. In addition, some of the current training opportunities are not accessible. Some programs limit participants through selective applications (e.g., few spots for fellows), targeted area of application (e.g., mental health), time commitment (e.g., semester-long course), and audience (e.g., researchers not practitioners) [4–6]. Further, the perception in the field of specific training programs producing “card-carrying” implementation scientists can exacerbate concerns about gatekeeping [9]. There is a need to continue building capacity in implementation science, with attention to the development of innovative programs that target a wider range of educational and accessibility needs, and to systematically evaluate the impact of such programs.

The Penn Implementation Science Institute (PennISI) is a novel implementation science training opportunity. At the time of its inception, the PennISI offered one of the first brief implementation science training programs, thus filling a needed gap in educational offerings [6]. As implementation science training offerings continue to develop, systematic evaluation of such efforts is needed to ensure rigor through achievement of specific competencies and understanding of strengths and weakness of programs. This paper describes the PennISI to offer a potential model for the field. Following the movement in the field to use established competencies [4, 5, 10–12], this study also evaluates the impact of the PennISI on advancing thematic implementation science competencies.

## Methods

Given the purpose of program evaluation, this project was deemed exempt by the Institutional Review Board at the University of Pennsylvania (Penn). The Consensus-Based Checklist for Reporting Survey Studies (CROSS) [13] guided the reporting of this evaluation (Additional File 1).

### Penn Implementation Science Institute

To help fill the gap in implementation science training, individuals at Penn developed the Penn Implementation Science Institute (PennISI). Briefly, the PennISI offered one of the first brief training programs through an institution other than the National Institutes of Health. Facilitated by the Penn Implementation Science Center (PISCE@LDI) and the Penn Master of Science and Health and Policy Research (MSHP) program, the PennISI aims to provide participants with the tools to design and execute rigorous implementation research. Course Directors developed the curriculum based on other exemplar training programs (e.g., Implementation Research Institute) and key foundational concepts in implementation science. To note, the leadership team included three faculty members including clinician scientists that balanced responsibilities between research, clinical work, and education, thus acknowledging the limited capacity to devote time solely to curriculum development. Launched in 2017, the PennISI originally hosted an in-person institute. Due to the COVID-19 pandemic, the institute pivoted to a virtual format, which increased capacity for participant attendance. Further, the curriculum evolved each year based on participants’ feedback and emerging areas in the field (e.g., health equity and implementation science).

Currently, the PennISI is intended for scholars at all career levels interested in learning more about the foundation of implementation science for application to future research. Applications are accepted on a first-come, first-served basis until capacity is reached. PennISI is a credit-bearing course; all participants pay for the equivalent of half of a credit unit with the option of enrolling into a full credit unit with additional assignments. Given the cost of attendance ($2950 in 2022), limited full and partial tuition scholarships are available for Penn affiliates and scholars from low- and middle-income countries. To earn a scholarship, participants must explain how the PennISI will contribute to their career development.

The 2022 PennISI included 4 days (20 h) of virtual programming. Through a combination of didactic lectures, small group discussion (randomly assigned at the start of PennISI but remain the same throughout the week), expert panels, and optional office hours (2 h daily), the institute covers both foundations of and advanced topics in implementation science (see Table 1 for a detailed description of the 2022 PennISI). Topics include the following: introduction to implementation science, models and frameworks, study design and methods, behavioral economics, global health application, health equity, implementation outcomes, implementation strategies, dissemination, quality improvement, grant writing, de-implementation, and implementation science in the real world (e.g., examples of research programs and stories of success in implementation science). The institute began with a keynote lecture by Dr. Wynne Norton, the National Cancer Institute Program Director of Implementation Science. Diverse Penn-based and external implementation science experts facilitate all sessions and small group discussions. Guided by the Consolidated Framework for Implementation Research [14], a novel activity completed in the small group discussion involves the collective brainstorming of barriers, facilitators, and implementation strategies for human papillomavirus vaccination implementation (see Additional File 2). To prepare for each day, participants read assigned journal articles and write brief discussion posts on the topics (see Additional File 3 for the list of “greatest hits” articles). No formal evaluation of knowledge retention is completed. All course activities (e.g., lectures and small group discussions) were facilitated via Zoom, an online videoconferencing platform, and Canvas, an online academic course platform. All course materials, recorded lectures, and supplemental content (e.g., additional readings) are available on Canvas for one month after the conclusion of the PennISI. Additional informal consultation related to participants’ project ideas occurs ad hoc. The 2022 PennISI included an additional 3-h debrief with the visiting global health participants facilitated by one of the core facilitators (A.E.V.P.). This manuscript evaluates the sixth annual PennISI in the summer of 2022 that enrolled 109 participants.

**Table 1:** Overview of PennISI components

Component	PennISI description
**Institution**	University of Pennsylvania (Penn) Penn Implementation Science Institute (PISCE) Master of Science and Health and Policy Research (MSHP) program
**Year**	2022 for institute reported in manuscript 2017 for inaugural PennISI
**Format**	The PennISI is a 4-day institute totaling 20 h (5 h/day). Facilitated via Zoom, the institute includes daily didactic or panel presentations and 45–60 min small group discussions. In addition, optional office hours are available 2 h per day. All participants pay for the equivalent of half of a credit unit with the option of enrolling into a full credit unit.
**Instructors**	All members of the learning team have expertise in implementation science.
	*Course directors:* **Rinad Beidas, PhD**- former founder and director of PISCE@LDI, Professor and Chair of Medical Social Sciences at Northwestern University **Meghan Lane-Fall, MD, MSHP**- current director of PISCE@LDI, Associate Professor of Anesthesiology and Critical Care and Epidemiology at Penn
	*Core faculty:* **Emily Becker-Haimes, PhD**- Assistant Professor of Psychiatry at Penn **Amanda Bettencourt, PhD**- Assistant Professor of Nursing at Penn **Chris Bonafide, MD, MSCE**- Associate Professor of Pediatrics at Penn, Associate Director of PISCE@LDI **Danielle Cullen, MD, MPH, MSHP-** Assistant Professor of Pediatric and Emergency Medicine at Penn and Children’s Hospital of Philadelphia **Rebecca Hamm, MD, MSCE**- Assistant Professor of Obstetrics and Gynecology at Penn **Katelin Hoskins, PhD, MSN, MBE**- Postdoctoral fellow at time of PennISI, current Assistant Professor of Nursing at Penn **Katharine Rendle, PhD, MSW, MPH**- Assistant Professor of Family Medicine and Community Health & Biostatistics, Epidemiology, and Informatics at Penn, Associate Director of PISCE@LDI **Sarita Sonalkar, MD, MPH**- Assistant Professor of Obstetrics and Gynecology at Penn **Rebecca Stewart, PhD**- Assistant Professor of Psychiatry at Penn **Amelia Van Pelt, PhD, MPH**- postdoc at PISCE@LDI at time of PennISI, current postdoc in Medical Social Sciences at Northwestern University
	*Visiting faculty:* **Srinath Adusumalli, MD, MSHP**- Assistant Professor of Clinical Medicine at Penn **Alison Buttenheim, PhD, MBA**- Associate Professor of Nursing and Health Policy at Penn, Scientific Director of the Center for Health Incentives and Behavioral Economics **Krisda Chaiyachati, MD, MPH, MSHP**- Physician Lead for Value-Based Care and Innovation of Verily Health Platforms **Kate Courtright, MD, MSHP**- Assistant Professor of Medicine, Pulmonary, and Critical Care at Penn **David Mandell, ScD**- Professor of Psychiatry at Penn, Director of Penn Center for Mental Health **Yehoda Martei, MD, MSCE**- Assistant Professor of Medicine at the Hospital of the University of Pennsylvania **Nathalie Moise, MD, MS**- Associate Professor of Medicine at Columbia University Irving Medical Center **Jen Myers, MD**- Professor of Clinical Medicine at Penn, Director of the Center for Health Care Improvement and Patient Safety **Michael Posencheg, MD**- Professor Clinical Pediatrics at Penn, Chief Medical Office at Penn Presbyterian Medical Center **Byron Powell, PhD, LCSW**- Associate Professor at Washington University, Associate Director for the Center for Dissemination and Implementation at WashU **Jonathan Purtle, DrPH, MSc**- Associate Professor of Public Health Policy at New York University, Director of Policy Research at NYU’s Global Center for Implementation Science **Rachel Shelton, ScD, MPH**- Associate Professor of Sociomedical Sciences at Columbia University **Rebecca Trotta, PhD, RN**- Director of Nursing Research and Science at Penn
	*Keynote speaker:* **Wynne Norton, PhD**- Program Director of Implementation Science in the Division of Cancer Control and Population Sciences at the National Cancer Institute
**Application process**	The PennISI is intended for scholars at all career levels interested in learning more about the foundation of implementation science for application to future research. Enrollment is open to all individuals (i.e., not exclusive to the Penn community). Applications open once per year for the annual institute. Applications do not require specific eligibility criteria. Interested participants submit an application via an online Penn platform, which are accepted on a first-come, first-served until capacity is reached.
**Trainees**	Full information about participant characteristics is provided in Table 2. Briefly, participants included researchers, practitioners, staff, and community partners. Training and areas of application varied
**Course topics**	The syllabus is available upon request before enrollment. Sessions incorporated various content areas to explain the following topics: Introduction to implementation science Models and frameworks Study design and methods Behavioral economics Global health application Health equity Implementation outcomes Implementation strategies Dissemination Quality improvement Grant writing De-implementation Implementation science in the real world (e.g., examples of research program and stories of success in implementation science)
**Assessment**	Participants do not take formal assessments. This is the first pre-post evaluation of participants’ competencies, but participants were not required to complete the surveys.
**Ongoing support**	In general, formal ongoing support is not provided upon conclusion of the PennISI. Course materials and session recordings are available to participants on the Canvas website for 1 month. Informal consultation has occurred upon request. The 2022 PennISI included an additional 3-h debrief session with the visiting global health scholars to review individual projects.

### Procedure

A pre-post survey design was used. Established educational competencies for dissemination and implementation research training programs [15] guided the development of the survey instruments. These competencies include 43 items grouped into four thematic domains (definition, background, and rationale; theory and approaches; design and analysis; and practice-based considerations) with four response options (no expertise in this area, beginner, intermediate, and advanced) (see Additional File 4 for each category and corresponding items). Example items per competency domain include (1) “Define what is and what is not D&I research” for definition, background, and rationale, (2) “Describe a range of D&I strategies, models, and frameworks” for theory and approaches, (3) “Identify and measure outcomes that matter to stakeholders, adopters, and implementers” for design and analysis, and (4) “Determine when engagement in participatory research is appropriate with D&I research” for practice-based considerations. These competencies are considered the “gold standard” for the evaluation of dissemination and implementation science training programs (4), so the use of these competencies ensured systematic, comprehensive evaluation and will enable future comparison across training programs. To note, these competencies did not inform the initial development of the PennISI curriculum, as they were identified post-hoc for use in the evaluation. Instruments were supplemented with items related to participant characteristics, including demographic information and prior experience with dissemination and implementation science (e.g., submission of related manuscript). Response options related to participants’ positions and areas in which participants apply implementation science were specified based on the team’s collective knowledge and experience. To elicit additional information about satisfaction with the PennISI, the post-survey included additional items provided by Penn to evaluate all university courses (e.g., “Overall rating/quality of course”) and open-ended questions eliciting feedback on the PennISI (e.g., “Please comment on any strengths, weaknesses or suggestions for future improvement.”). The final pre-survey and post-survey instruments included 55 and 58 items, respectively (Additional File 4).

All surveys were administered via REDCap [16]. To maintain confidentiality, participants were not required to provide identifying information (e.g., name or email). In addition, neither the pre-survey nor the post-survey were required. To strongly encourage participation, the pre-survey distribution email included the following description: “The purpose is to allow us to evaluate the effectiveness of our program.” The pre-survey was distributed 4 days and again one day before the start of the PennISI, and the post-survey was distributed on the last day of the PennISI with a follow-up reminder at 12 days post PennISI.

### Data analysis

Records that included data for any of the survey items were included in the analysis, and missing data were not imputed (i.e., the number of records in each calculation may differ depending on the response rate for that individual item). Duplicate participant entries were deleted when identifiable information was provided. Participant affiliations and participant positions were condensed into thematic categories (e.g., universities and academic medical centers representing the “US-based academic institution” category; doctoral student and undergraduate student representing the “pre-doctoral” category; master’s students were separated given the participation of doctoral-level clinical fellows enrolled in master-level programs at University of Pennsylvania). Given the self-reported nature of the questions, some respondents’ specified positions in the “other” category may have overlapped with existing categories, but reported items were preserved rather than grouped into categories. To calculate the mean scores for each of the competencies, the response options were coded from 0 to 4 (i.e., no expertise in this area — advanced). Mean composite scores were created for each of the competency domains by pooling data for all items within respective categories (e.g., 10 items for definition-related theme reflected in respective mean score). Descriptive statistics on the pre-survey and post-survey data were calculated. All analyses were completed in Stata Statistical Software (15.1; College Station, 2017). Given brief responses, an informal inductive analysis approach was conducted on the open-ended responses in which prevalent themes were identified by one investigator (A.E.V.P.).

## Results

### Participant characteristics

Nearly all participants completed the pre-survey (95.41%) (Table 2). Notably, the cohort included a racially diverse group of participants with 33.65% of participants identifying as White, 15.38% as Black or African American, 14.42% as Asian, 1.92% as American Indian or Alaskan Native, and 1.92% as Native Hawaiian or Other Pacific Islander. The majority of individuals were affiliated with an academic institution based in the USA (87.76%), and faculty (Assistant Professor, Associate Professor, and Professor) comprised approximately half of the cohort (47.11%). Additional participant positions varied, including academic trainees (e.g., postdoctoral fellow and doctoral student), clinicians (e.g., pediatrician), academic-based staff (e.g., research staff), and community partners (e.g., state health department). Fourteen attendees received a scholarship for participation, four of which were participants from low- and middle-income countries. Most participants classified their implementation science experience level as novice (24.04%) or beginner (57.69%), and two individuals reported expert-level experience. The content area in which participants apply implementation science was distributed fairly equally, but behavioral and mental health was the most common area of application (27.88%). Eighteen participants indicated that they had not yet applied implementation science to their research. Further, most participants had not yet submitted an implementation science-related grant (74.04%). Among those with implementation science experience, 21 individuals had submitted a related manuscript. Fifty-five participants completed the post-survey (response rate overall = 50.46%), and the demographic characteristics reflected those of the pre-survey respondents (Table 2) (e.g., greatest proportion of respondents in post-survey were affiliated from US-based academic institutions, specifically assistant professors). Post-surveys did not collect information about experience with D&I.

**Table 2:** Respondent characteristics

	**Pre** ***N* (%)**	**Post** ***N* (%)**
**Demographics**		
**Total**	104	55
**Affiliation**^**a**^		
Government	5 (5.10)	2 (4.08)
International-based academic institution	4 (4.08)	5 (10.20)
Not-for-profit organization	5 (5.10)	1 (2.04)
US-based academic institution^b^	86 (87.76)	41 (83.67)
**Position**		
Professor	2 (1.92)	2 (3.64)
Associate professor	12 (11.54)	4 (7.27)
Assistant professor	35 (33.65)	22 (40.00)
Postdoctoral fellow	9 (8.65)	5 (9.09)
Pre-doctoral trainee	1 (0.96)	0
Master’s student	3 (2.88)	1 (1.82)
Research staff	13 (12.50)	7 (12.73)
Non-faculty researcher	2 (1.92)	2 (3.64)
Clinician	13 (12.50)	6 (10.91)
Community partner	1 (0.96)	1 (1.82)
Other	13 (12.50)	5 (9.09)
**Race**^**a**^		
American Indian or Alaskan Native	2 (1.92)	1 (1.82)
Asian	15 (14.42)	10 (18.18)
Black or African American	16 (15.38)	10 (18.18)
Native Hawaiian or Other Pacific Islander	2 (1.92)	2 (3.64)
White	69 (66.35)	33 (60.00)
**Hispanic or Latinx**		
Yes	6 (5.88)	2 (3.77)
No	96 (94.12)	51 (96.23)
**Experience with D&I**		
**D&I experience level**		
Novice	25 (24.04)	
Beginner	60 (57.69)	
Intermediate	17 (16.35)	
Expert	2 (1.92)	
**D&I content areas**^**a**^		
Acute care	19 (18.27)	
Behavioral/mental health	29 (27.88)	
Cancer	17 (16.35)	
Cardiovascular disease	9 (8.65)	
Global health	13 (12.50)	
HIV	9 (8.65)	
Pediatrics	26 (25.00)	
Other	28 (26.92	
Not yet applied	18 (17.31)	
**D&I grant submission**		
Yes	27 (25.96)	
No	77 (74.04)	
**D&I grant funded**		
Yes	21 (77.78)	
No	6 (22.22)	
**D&I manuscript submission**		
Yes	21 (95.45)	
No	1 (4.55)	
**D&I manuscript published**		
Yes	21 (95.45)	
No	1 (4.55)	

^a^Check all that apply

^b^Among participants who provided information about affiliation, 34 were affiliated with the University of Pennsylvania or the Children’s Hospital of Philadelphia

### Overall impact

Overall, participants indicated high value from the PennISI, as all course evaluation criteria scored above a mean score of 3.55 (median score 4.00; 4.00 as highest possible score) (Additional File 5). The commitment of the course directors received the highest score with an almost perfect rating (mean score of 3.98; median score of 4.00). The overall rating and quality of the course had a mean of 3.77 (median score of 4.00). Further, participants reported high educational value and amount of information learned with a mean score of 3.68 (median score of 4.00). The item with the lowest mean score related to the appropriateness and challenge of the workload and material (mean score of 3.55; median score of 4.00).

### Implementation science competencies

At baseline, mean scores for all of the implementation science competency domains were below beginner-level (i.e., composite score < 1) (Table 3). In the pre-survey, participants reported the highest level of knowledge related to definition, background, and rationale (mean score 0.82; median score 0.80), followed by practice-based competencies (mean score 0.69; median score 0.58), theory and approaches (mean score 0.66; median score 0.64), and design and analysis (mean score 0.54; median score 0.43). The individual competency with the greatest proportion of participants reporting “no expertise in this area” related to concepts of de-adoption and de-implementation study design (70.59%) (Additional File 6). Conversely, competencies with the highest baseline level of knowledge related to the impact of disseminating, implementing, and sustaining effective interventions as well as the importance of incorporating perspectives from different stakeholder groups (20.59% indicating no expertise for each of the two items). On the other end of the spectrum, four participants indicated advanced-level knowledge on various competency items (e.g., “Differentiate between D&I research and other related areas, such as efficacy research and effectiveness research”) (Additional File 6).

**Table 3:** Pre-post scores for implementation science competencies by theme

	**Mean (range)**	
	Pre	Post
D&I definition, background, and rationale	0.82 (0–2.60)	1.51 (0.30–2.60)
D&I theory and approaches	0.66 (0–2.29)	1.41 (0.14–2.14)
D&I design and analysis	0.54 (0–2.21)	1.30 (0.14–2.21)
D&I practice-based considerations	0.69 (0–2.67)	1.43 (0–2.25)

Mean scores for all implementation science competency domains increased in the post-survey to a score between beginner-level and intermediate-level expertise (i.e., composite score between 1 and 2) (Table 3). Similar to the baseline data, participants reported the highest level of knowledge related to definition, background, and rationale (mean score 1.51; median score 1.50), followed by practice-based competencies (mean score 1.43; median score 1.33), theory and approaches (mean score 1.41; median score 1.43), and design and analysis (mean score 1.30; median score 1.21). All individual items had seven or fewer participants indicating no expertise in the specific competency (range = 0–7), with the majority of items reflecting advanced-level competencies per the Padek taxonomy (Additional File 6). Items related to identifying and measuring outcomes as well as identifying the potential impact of disseminating, implementing, and sustaining effective interventions both reported all participants with at least beginner-level expertise (Additional File 6). One participant reported an inability to define what is and what is not D&I research. Acknowledging the bias of the smaller response rate in the post-survey, participants indicated advanced-level knowledge in 22 individual competency items (e.g., “Determine when engagement in participatory research is appropriate with D&I research”).

### Qualitative themes

Table 4 provides an overview of and illustrative quotations for the themes that emerged from the participants’ post-survey open-ended responses.

**Table 4:** Post-survey qualitative themes

Theme	Sample ouotes
Overall impressions	“The energy of the facilitators, course leaders, and guest speakers was next level and infectious. It really helped with motivation.”
	“This course was incredibly helpful and I’m so grateful to have participated this summer. I attained many of my goals to learn more about the terminology and how to apply it to my own research.”
PennISI content	“As a relative novice, I at times felt some of the lectures were too advanced for more and were focused more on those writing an NIH grant using implementation science. It would have been nice to hear more about smaller scale uses of implementation science.”
	“I particularly liked the health equity seminar and the succeeding in implementation research panel.”
PennISI structure	“The virtual format was very convenient, especially for someone with a young child and for those in other countries. However, in-person institutes/workshops are always more energizing and engaging for me, even though the leaders did a fantastic job engaging attendees in a virtual format.”
	“I think the combination of lectures (with ability to ask questions” and small group sessions worked very well. Overall this was an outstanding training session.”
Suggestions for improvement	“It may be helpful to incorporate a ‘Terminology Toolkit’ that provides definitions to some of the key terminology that will be used throughout the institute. It may be helpful for those with limited knowledge to more easily follow along with talks throughout the week.”
	“The course might be strengthened by adding workshops (practicing some of the most basic concepts) to the small groups. Small group discussions were helpful and facilitators were great, but more structure would be helpful.”
	“I wonder if the organizers will consider having some breakout sessions based on experience level, as being in a group with [multiple] funded investigators who were also actively doing D&I research (and were very talkative) made me really not want to participate in any group activities or make any commentary because I felt totally out of my depth at times.”

#### Overall impressions

Most participants expressed positive opinions of the PennISI overall. Respondents described the training program as “phenomenal” and “amazing.” Many of the responses attributed the value of the course to the PennISI instructors, specifically their enthusiasm, expert knowledge, and commitment to the program. Although the majority of participants reported high value from the PennISI, opinions varied depending on participants’ prior level of implementation science knowledge and experience; described further in the next sections.

#### PennISI content

Participants commented on different aspects of the content included in the PennISI. With regard to the didactic topics, respondents appreciated the inclusion of a focus on health equity and applied examples; some participants desired more opportunities for application. However, opinions on the content overall varied. Some participants found the material too introductory, while others perceived the material as too advanced for an introductory implementation science training program. Related, a common theme related to the appropriateness of the material for the intended level of the audience emerged. For those new to the field of implementation science, participants felt overwhelmed by the amount and level of material and indicated a sense of the target audience being future grant applicants. As a result, some respondents expressed limitations in engagement, value gained from the institute, and experience of trying to keep up with the material.

#### PennISI structure

Participants discussed various components of the PennISI structure. Overall, participants appreciated the virtual nature of the institute because the format increased accessibility and facilitated the provision of course materials online. However, some participants expressed that a virtual institute cannot fully replace engagement in an in-person program. To address engagement, respondents praised the communication of the PennISI, specifically the amount and quality of communication from the instructors, level of engagement in the Zoom chat, and the ability to ask questions throughout the institute (e.g., in lectures, office hours, and small group discussions). Further, although participants appreciated the inclusion of the office hours, some participants reported scheduling conflicts preventing attendance or feeling rushed in the session. Finally, opinions on the small group discussions varied. Overall, participants seemed to prefer the discussion that incorporated a case example (e.g., activity to identify CFIR determinants in group) and appreciated the opportunity to clarify course content. However, perceptions on the value gained and opinion on the structure of the small group discussions varied. Some participants felt that the range of implementation science experience and knowledge in the group, as well as stage of career, influenced the engagement of participants in these sessions (e.g., more knowledgeable participants dominated the conversation).

#### Suggestions for improvement

Participants suggested various ideas for improving the PennISI in the future, most of which related to the diverse implementation science experience level. First, participants recommended dividing the PennISI into two institutes: introduction to implementation science and advanced topics in implementation science. In the absence of creating two separate institutes, some participants requested extending the PennISI to a longer period of time to enable greater processing of information (e.g., 2 weeks to allow synthesis and reflection). Second, participants offered suggestions to modify the small group discussions. Most commonly, respondents recommended splitting the groups based on level of experience or knowledge with implementation science. In addition, some participants proposed creating more structure in the sessions through the use of case studies or opportunities for application of the didactic topic. Third, recommendations surrounded the didactic content. Some participants recommended providing a stronger foundation in implementation science at the beginning of the institute through the provision of a reference terminology dictionary, for example. Related, some respondents articulated a desire for less-complex implementation science project examples to illustrate a feasible place to start for novice implementation researchers. In addition, some participants with a higher level of experience requested specific lecture topics (e.g., adaptation).

## Discussion

There is a critical need to build capacity in implementation science to ensure the future growth of the field. This study described and assessed the sixth annual PennISI, an innovative 4-day virtual implementation science institute. Findings indicate high value gained and perspectives on successful programmatic components, which can serve as a model for other brief training programs. Further, the evaluation provides valuable insight for improving future implementation science training programs, such as modifying course content and structure to align with participants’ baseline implementation science knowledge (see Table 5 for an overview of recommendations for future training programs).

**Table 5:** Recommendations for brief implementation science training programs

**Recommendation**
**Participants**
Target individuals seeking introductory implementation science skills
Provide financial support for colleagues from LMICs
**Pre-course**
Communicate intended audience
Share course syllabus at time of registration
Provide written course goals and competencies
**Structure**
Virtual format to increase access
Separate participants into thematic groups for small group discussion (e.g., level of implementation science expertise or career level)
Create two institutes: one for foundational information and one for advanced topics^a^
**Content**
Incorporate interactive elements (e.g., group discussion, office hours, course chat)
Include case examples for application of topics
Include session on grant writing for academic participants
Make course materials open source

^a^Resources and feasibility permitting

Overall, participants reported that the PennISI provided a successful implementation science training program. The high scores on all post-evaluation competencies highlight substantial perceived knowledge gained from the institute and the potential to yield change in all thematic domains. Participants’ highest ratings for competencies related to definition, background, and rationale suggest that brief training programs may particularly have the opportunity to impact introductory skills. Further, the positive perception of the PennISI from the majority of participants demonstrates the acceptability of the institute structure. Specifically, facilitating a virtual format increases the accessibility for participants. Although virtual programs cannot replace the dynamic fostered by in-person programs, the format creates a more inclusive institute to help extend reach to a diverse set of learners. Additional strengths of the PennISI included the inclusion of interactive elements, such as applied examples in lectures, small group discussions, office hours, and attention to questions in the chat. These preferences highlight the value of facilitating activities to foster engagement and create a positive learning environment. These findings are generalizable beyond the PennISI, and this institute can serve as a model for the development of other brief, virtual implementation science training programs, as well as other rapidly emerging areas of interest (e.g., climate change and artificial intelligence research).

This evaluation contributes to the efforts to systematically assess implementation science capacity building initiatives, which is critical for ensuring the rigor of implementation science education [4, 5, 10]. A strength of this study involved the use of established competencies for evaluating implementation science training programs [15]. This approach enables comparison of training programs on standard measures, both within the PennISI and outside of the institution. Perhaps most importantly, fostering cross-institutional discussion related to building capacity will advance the field of implementation science. Implementation science exists to close research-to-practice gaps. Educational initiatives ought to leverage this goal by not recreating the wheel through the development of new implementation science training programs de novo but rather by learning from existing effective courses. Standardized evaluation provides insight into what does and does not work for programs. The field has begun engaging in collaborative efforts through the development of a repository for implementation science-related grants [17] and sharing of translated frameworks, for example. Training programs ought to consider making course materials open source as well; in addition to the materials provided in the Additional Files, PennISI materials are available upon reasonable request to Course Directors. However, greater investment in capacity building is required to make this approach feasible and appealing to instructors often operating within the constraints of academia. Collaboration will shift the effort to help ensure that training programs achieve desired implementation science competencies.

Further, this study highlighted the type of learners seeking out brief implementation science training. The PennISI included a cohort of individuals with a diverse range of implementation science experiences and knowledge, ranging from novice to expert at baseline. These findings indicate that programs are attracting a mixed group of learners, which can result in both benefits (e.g., peer-to-peer learning) and limitations. For example, the institute experienced some challenges in meeting the needs of all participants, as discussed in this study and consistent with other capacity building initiatives [11]. This issue warrants thoughtful consideration for tailoring programs. Further, this study did not evaluate change in overall implementation science experience level in the post-survey, as a 4-day institute would not expect to yield meaningful change in overall expertise. Change in individual competency items is expected, but the overall level of knowledge (i.e., from beginner-level implementation scientist to intermediate-level implementation scientist) often requires increased time and application of concepts beyond 20 h of learning. Therefore, brief training programs may cater most appropriately to the first two phenotypes of learners who desire increased awareness and basic understanding of the field for collaboration but require additional mentorship for independent implementation research [9]. In addition, the majority of participants included individuals in academia, specifically faculty. Consistent with other capacity building efforts [4, 5], this trend helps guide the inclusion of topics for training. For example, given the nature of the audience, programs might include a session on writing grants in implementation science, both as a collaborator and a project lead. The dominance of academic researchers indicates that capacity building needs to improve recruitment for other individuals crucial to success in implementation science, such as practitioners who are often the minority in training programs [5, 6]. Training programs have begun to address this need (e.g., The Center for Implementation certificate program). One strategy could include advertisement of the training institute with researchers’ community partners. In addition to practitioners, this study emphasized the need to recruit global health colleagues. The PennISI included a cohort of individuals from four countries (United States, Ghana, South Africa, and Tanzania), which increases the diversity of the implementation science community. However, given that a great deal of implementation science capacity building occurs in high-income countries, training programs should increase access to global health colleagues in low- and middle-income countries through continued financial support and sponsorship with global health centers, as done for the PennISI. For sustainability, efforts should pivot to long-term partnership with global institutions to facilitate locally led training programs [18, 19]. As programs continue to develop, understanding the characteristics of individuals seeking out training will help tailor programs and recruitment accordingly.

In addition to informing tailoring of programs related to participants, findings from this evaluation provide insights for improving the structure of implementation science capacity building efforts. The evaluation revealed the potential impact of a mismatch in a training program’s course materials and participants’ level of implementation science knowledge (i.e., too advanced or too beginner depending on the participant). To address the demand for implementation science training, programs have emerged to try to meet the needs of all learners. The PennISI was advertised as providing both foundational and advanced topics in implementation science. However, survey-style programs that incorporate a lecture on each of the key topics in the field can limit the ability to provide in-depth information on each topic, which could result in confusion for novices or repetition for advanced scholars. To address this, course directors should clearly communicate the intended audience (e.g., early-career researchers) and level of information covered (e.g., introduction to implementation science) through written goals and competencies so potential participants have a better understanding of the anticipated material. If feasible, instructors ought to consider sharing the course syllabus at the time of registration for increased transparency. In the absence of resource constraints, the creation of multiple implementation science institutes that cater to different audiences would better address the various needs of learners. For example, dividing programs into two institutes, one for introductory material and one for advanced topics, could help standardize the audience and foster better engagement, knowledge retention, and satisfaction among participants. This modification could help address the lower mean score for complicated competencies (e.g., design and analysis) and participants’ desire for specific lecture topics. Given the limited bandwidth and resources to facilitate multiple institutes, one strategy to modify existing structures could involve separating participants into thematic groups for small group discussion (e.g., level of implementation science expertise or career level). To facilitate this process, instructors should collect and analyze self-reported information related to participants’ experience before the start of the training program. Further, the creation of multiple institutes (e.g., one beginner-level and one advanced-level, or simply offering of two beginner institutes) would increase access limited by enrollment constraints in a single institution. This evaluation suggests that application increases learning. Courses should incorporate more case examples that enable participants to apply concepts learned in didactic sessions (e.g., presenting the implementation science logic model in the beginning of the institute and revisiting the model with the introduction of each new concept; participants could complete the model concurrently for their project ideas). Participants could engage in these activities at their level of expertise and interest. Finally, to effectively complete all of the aforementioned recommendations to improve future training programs, greater institutional support, such as dedicated administrative support (0.5 FTE or greater depending on the volume of registrants) is necessary.

This evaluation has some limitations. First, although consistent with non-incentivized surveys, the 50% response rate for the post-survey may have introduced non-response bias. Second, the PennISI was facilitated via a virtual platform. Therefore, the level of engagement with the course may have varied among participants (e.g., participants not attending sessions or participants splitting attention), which could have influenced knowledge retention and the reported post-survey outcomes. Determination of complete engagement of participants at each session is not feasible. Participants who did not complete the survey may not have perceived as much value gained from the PennISI, which could have positively skewed the change in competencies. Third, the post-survey measures were obtained immediately after the conclusion of the PennISI, which limited the ability to assess change in competencies over time. Future efforts could consider a longitudinal design to assess sustained knowledge retention and application to research. Fourth, the free-response question format limited the depth of qualitative information obtained. Although the data provided helpful insights, future efforts could conduct in-depth approaches with a sub-set of participants to elicit more detailed input. Fifth, the D&I competencies included in the evaluation did not inform the development of the PennISI curriculum, which could explain the lack of change in some of the post-survey items. Future efforts could modify the curriculum based on targeted competencies to assess changes in significance of pre-post survey differences.

## Conclusions

This study provides an example of an effective training institute on advancing implementation science competencies that can serve as a model for brief, virtual training programs. Findings also highlight insights for improving future programs, which emphasizes the value of standardized evaluation of educational programs. Efforts should continue to refine and adapt capacity building to meet the needs of the growing, heterogenous implementation science community.

### Supplementary Information

**Additional file 1. ** Checklist for Reporting of Survey Studies (CROSS).

**Additional file 2. **CFIR Small Group Activity.

**Additional file 3. **Greatest Hits Articles Provided to Participants.

**Additional file 4. **Pre- and Post-survey Instruments.

**Additional file 5. **Penn Course Evaluation.

**Additional file 6. **Individual Implementation Science Competencies by Theme.

## Data Availability

All data is provided in the Additional Files, and additional materials are available upon reasonable request.

## Abbreviations

PISCE@LDI: Penn Implementation Science Center
PennISI: Penn Implementation Science Institute
