# Supplementary material for: Evaluation of a brief virtual implementation science training program: the Penn Implementation Science Institute
Source: Implement Sci Commun. 2023 Nov 6;4:131. doi: 10.1186/s43058-023-00512-5 (PMC10626776; doi:10.1186/s43058-023-00512-5)
Supplement: Supplementary file 2 — Additional file 2. CFIR Small Group Activity. [file 43058_2023_512_MOESM2_ESM.docx]

**Additional File 2. CFIR Small Group Activity.**

**Background.**

Human papillomavirus vaccine (HPV) is a common sexually transmitted infection among United States adults. Approximately 80 million Americans are infected with HPV, and annually another 14 million become newly infected.

**The evidence-based practice (EBP).**

The HPV vaccine, which is effective and safe, has been available in the United States since 2006, yet vaccination rates remain low. The Healthy People 2020 target for completion of the three dose HPV vaccination series is 80% for adolescents aged 13-15. In 2018, 70% of girls aged 13017 had received at least one HPV vaccine dose, but only 54% had received three doses. Coverage for boys was even lower, with only 66% receiving one dose and 49% receiving three doses.

**Task.**

You’ve been asked to provide an implementation consultation for a primary care clinic. The clinic is a Federally Qualified Health Center that serves a low-income neighborhood in West Philadelphia. The clinic is trying to boost its HPV vaccination rates. Currently, the vaccination rate for eligible children and teenagers is hovering around 50%. The clinic wants to boost it to at least 80%.

Your small group will ask contextual questions to better understand how to get this clinic to boost its vaccination rate. Consider the CFIR domains Outer Setting, Inner Setting, and Characteristics of Individuals as they relate to this clinic. What additional information do you need to fully develop a sense of what’s going on in the clinic? Who do you need to talk to? How will you go about collecting these data?

**Assignment.**

1. Divide into four groups to tackle 3 of 5 CFIR domains (Individuals involved, Inner setting, Outer setting).
2. Select a spokesperson.
3. Don’t get hung up on setting details; make them up if needed.
4. Feel free to access the CFIR wiki.
