# Supplementary material for: Evaluation of a brief virtual implementation science training program: the Penn Implementation Science Institute
Source: Implement Sci Commun. 2023 Nov 6;4:131. doi: 10.1186/s43058-023-00512-5 (PMC10626776; doi:10.1186/s43058-023-00512-5)
Supplement: Supplementary file 3 — Additional file 3. Greatest Hits Articles Provided to Participants. [file 43058_2023_512_MOESM3_ESM.docx]

**Additional File 3. Greatest Hits Articles Provided to Participants.**

# Penn Implementation Science 2022 Institute | Greatest Hits Reading List

The following list of resources is meant to supplement your learnings from the Penn Implementation Science Institute. It is not all-inclusive; rather, it represents works that explain core IS concepts. Feel free to contact the Institute directors or individual speakers for more references relating to topics of interest.

# Suggested Textbook:

Brownson RC, Colditz GA, Proctor EK. Dissemination and Implementation Research in Health: Translating Science into Practice (2nd ed). New York, NY: Oxford University Press; 2017.

| **Citation** | **Comments** |
| --- | --- |
| **Overview** | |
| 1. Eccles MP, Mittman BS. [Welcome to](https://implementationscience.biomedcentral.com/articles/10.1186/1748-5908-1-1) [implementation science.](https://implementationscience.biomedcentral.com/articles/10.1186/1748-5908-1-1) *Implement Sci.* 2006;1:1. | This commentary introduced the journal Implementation Science and includes definitions and a brief history of the field. |
| 2. Bauer M, Damschroder L, Hagedorn H, Smith J, Kilbourne A. [An introduction to implementation](https://bmcpsychology.biomedcentral.com/articles/10.1186/s40359-015-0089-9/open-peer-review) [science for the non-specialist.](https://bmcpsychology.biomedcentral.com/articles/10.1186/s40359-015-0089-9/open-peer-review) *BMC Psychology*. 2015;3(32):1-12. | This review defines implementation science, distinguishes it from interventional research, explains the importance of frameworks, theories, and models, and reviews study designs used in implementation science research studies. |
| 3. Curran GM. [Implementation science made too](https://implementationsciencecomms.biomedcentral.com/articles/10.1186/s43058-020-00001-z) [simple: a teaching tool.](https://implementationsciencecomms.biomedcentral.com/articles/10.1186/s43058-020-00001-z) Implementation Science Communications. 2020 Dec;1(1):1-3. | This teaching tool simply describes implementation science. |
| 4. Lane-Fall MB, Curran GM, Beidas RS. [Scoping](https://bmcmedresmethodol.biomedcentral.com/articles/10.1186/s12874-019-0783-z) [implementation science for the beginner:](https://bmcmedresmethodol.biomedcentral.com/articles/10.1186/s12874-019-0783-z)  [locating yourself on the “subway line” of](https://bmcmedresmethodol.biomedcentral.com/articles/10.1186/s12874-019-0783-z) [translational research.](https://bmcmedresmethodol.biomedcentral.com/articles/10.1186/s12874-019-0783-z) BMC medical research methodology. 2019 Dec;19(1):133. | This teaching tool helps you place your research questions on the “implementation science subway line.” |
| 5. Kilbourne AM, Glasgow RE, Chambers DA. [What Can Implementation Science Do for You?](https://pubmed.ncbi.nlm.nih.gov/33107001/) [Key Success Stories from the Field.](https://pubmed.ncbi.nlm.nih.gov/33107001/) Journal of General Internal Medicine. 2020 Nov;35(2):783-7 | This paper offers examples of success stories from implementation science. |

| **Citation** | **Comments** |
| --- | --- |
| 6. Proctor, E, Geng, E. [A new lane for science.](https://pubmed.ncbi.nlm.nih.gov/34735221/) Science. 2021 Nov; 374 (6568): 659. | This paper is an influential editorial calling out the critical nature of the field. |
|  | |
| **Frameworks** | |
| 7. Nilsen, P. [Making sense of implementation](https://implementationscience.biomedcentral.com/articles/10.1186/s13012-015-0242-0) [theories, models and frameworks.](https://implementationscience.biomedcentral.com/articles/10.1186/s13012-015-0242-0) *Implement Sci.* 2015;10:53 | This review acknowledges that there are many frameworks, theories, and models (FTMs) used in implementation science and establishes a three-part taxonomy of these FTMs: process models, determinant frameworks, and evaluation models. |
| 8. Damschroder LJ, Aron DC, Keith RE, Kirsh SR, Alexander JA, Lowery JC. [Fostering](https://implementationscience.biomedcentral.com/articles/10.1186/1748-5908-4-50) [implementation of health services research](https://implementationscience.biomedcentral.com/articles/10.1186/1748-5908-4-50) [findings into practice: a consolidated framework](https://implementationscience.biomedcentral.com/articles/10.1186/1748-5908-4-50) [for advancing implementation science.](https://implementationscience.biomedcentral.com/articles/10.1186/1748-5908-4-50) *Implement Sci*. 2009;4:50. | This paper introduced what is now one of the most popular frameworks in implementation science: CFIR (pronounced “SEE-fur”), the Consolidated Framework for Implementation Research. Note, CFIR 2.0 is forthcoming. |
| 9. Tabak RG, Khong EC, Chambers DA, Brownson RC. [Bridging research and practice: models for](https://www.ncbi.nlm.nih.gov/pmc/articles/PMC3592983/) [dissemination and implementation research.](https://www.ncbi.nlm.nih.gov/pmc/articles/PMC3592983/) Am J Prev Med. 2012;43:337-350. | This paper discusses the utility of frameworks, theories, and models (FTMs) in implementation science and offers an inventory of FTMs. Consider reading this paper after the Per Nilsen paper (#6 in this list). |
| 10. Strifler L, Cardoso R, McGowan J, Cogo E, Nincic V, Khan PA, Scott A, Ghassemi M, MacDonald H, Lai Y, Treister V. [Scoping review](https://www.ncbi.nlm.nih.gov/pubmed/29660481) [identifies significant number of knowledge](https://www.ncbi.nlm.nih.gov/pubmed/29660481) [translation theories, models, and frameworks with](https://www.ncbi.nlm.nih.gov/pubmed/29660481) [limited use.](https://www.ncbi.nlm.nih.gov/pubmed/29660481) Journal of clinical epidemiology. 2018 Aug 1;100:92-102. | This paper provides an update to the literature on the current inventory of FTMs. |
|  | |
| **Design** | |
| 11. Brown CH, Curran G, Palinkas LA, Aarons GA, Wells KB, Jones L, Collins LM, Duan N, Mittman BS, Wallace AM, Tabak RG, Ducharme L, Chambers D, Neta G, Wiley T, Landsverk J, Cheung K, Cruden G. [An overview of research](https://www.ncbi.nlm.nih.gov/pmc/articles/PMC5384265/) [and evaluations designs for dissemination and](https://www.ncbi.nlm.nih.gov/pmc/articles/PMC5384265/) [implementation.](https://www.ncbi.nlm.nih.gov/pmc/articles/PMC5384265/) *Annual Review of Public Health.* 2017;38:1-22. | This paper is a broad overview of research designs relevant to implementation science, discussing challenges including threats to validity. Quasi- experimental, randomized trials, and adaptations of randomized trials (e.g., roll-out randomized designs) are discussed. |

| **Citation** | **Comments** |
| --- | --- |
| 12. Curran GM, Bauer M, Mittman B, Pyne JM, Stetler C. [Effectiveness-implementation hybrid](https://www.ncbi.nlm.nih.gov/pmc/articles/PMC3731143/) [designs: combining elements of clinical](https://www.ncbi.nlm.nih.gov/pmc/articles/PMC3731143/) [effectiveness and implementation research to](https://www.ncbi.nlm.nih.gov/pmc/articles/PMC3731143/) [enhance public health impact.](https://www.ncbi.nlm.nih.gov/pmc/articles/PMC3731143/) *Med Care.* 2012;50:217–226. | This paper introduces the concept of “hybrid trials” that have a dual focus: intervention effectiveness and implementation strategy. There are three types of hybrid trials detailed in this paper. |
| 13. Wolfenden L, Foy R, Presseau J, Grimshaw JM, Ivers NM, Powell BJ, Taljaard M, Wiggers J, Sutherland R, Nathan N, Williams CM[.](https://www.bmj.com/content/372/bmj.m3721) [Designing and undertaking randomised](https://www.bmj.com/content/372/bmj.m3721) [implementation trials: guide for researchers.](https://www.bmj.com/content/372/bmj.m3721) bmj. 2021 Jan 18;372. | This paper provides a broad overview of designing and deploying randomized implementation trials from an international author group. |
|  | |
| **Methods** | |
| 14. Palinkas LA, Mendon SJ, Hamilton AB. [Innovations in Mixed Methods Evaluations.](https://www.ncbi.nlm.nih.gov/pubmed/30633710) Annual review of public health. 2019 Apr 1;40. | Implementation science relies heavily on the use of mixed methods (qualitative + quantitative) approaches to inquiry. This article reviews the use of mixed methods in IS research and explains different types of mixed methods research. |
|  | |
| **Outcomes** | |
| 15. Proctor E, Silmere H, Raghavan R, Hovmand P, Aarons G, Bunger A, Griffey R, Hensley M. [Outcomes for implementation research:](https://www.ncbi.nlm.nih.gov/pmc/articles/PMC3068522/) [conceptual distinctions, measurement challenges,](https://www.ncbi.nlm.nih.gov/pmc/articles/PMC3068522/) [and research agenda.](https://www.ncbi.nlm.nih.gov/pmc/articles/PMC3068522/) *Adm Policy Ment Health*. 2011; 38(2):65-76. | This seminal paper introduces and defines eight implementation outcomes: acceptability, adoption, appropriateness, costs, feasibility, fidelity, penetration, and sustainability. |
| 16. Weiner BJ, Lewis CC, Stanick C, Powell BJ, Dorsey CN, Clary AS, Boynton MH, Halko H[.](https://implementationscience.biomedcentral.com/articles/10.1186/s13012-017-0635-3) [Psychometric assessment of three newly](https://implementationscience.biomedcentral.com/articles/10.1186/s13012-017-0635-3) [developed implementation outcome measures.](https://implementationscience.biomedcentral.com/articles/10.1186/s13012-017-0635-3) [Implementation Science.](https://implementationscience.biomedcentral.com/articles/10.1186/s13012-017-0635-3) 2017 Dec;12(1):108. | This paper provides psychometric properties of 3 brief pragmatic measures of implementation outcomes |
| 17. Damschroder L, Reardon, C, Widerquist M, Lowery, J. [Conceptualizing outcomes for use with](https://pubmed.ncbi.nlm.nih.gov/35065675/) [the CFIR: the CFIR outcomes addendum.](https://pubmed.ncbi.nlm.nih.gov/35065675/) 2022 January. | This paper provides conceptual distinctions between types of outcomes that are appropriate to be used with CFIR and other determinant implementation frameworks. |

| **Strategies** | |
| --- | --- |
| 18. Powell BJ, McMillen JC, Proctor EK, et al. [A](https://www.ncbi.nlm.nih.gov/pmc/articles/PMC3524416/) [compilation of strategies for implementing clinical](https://www.ncbi.nlm.nih.gov/pmc/articles/PMC3524416/) [innovations in health and mental health.](https://www.ncbi.nlm.nih.gov/pmc/articles/PMC3524416/) *Med Care Res Rev.* 2012;69:123-157. | This article discusses six categories of implementation strategies: Planning, Education, Financing, Restructuring, Quality Management, and Policy.  The authors recently published an update to their 2012 paper, but this earlier paper may be more accessible for people new to IS. (The citation for the newer paper is Powell et al, [A refined compilation of implementation](https://implementationscience.biomedcentral.com/articles/10.1186/s13012-015-0209-1) [strategies.](https://implementationscience.biomedcentral.com/articles/10.1186/s13012-015-0209-1) *Implement Sci*. 2015; 10:21.) |
| 19. Waltz TJ, Powell BJ, Fernández ME, Abadie B, Damschroder LJ. [Choosing implementation](https://implementationscience.biomedcentral.com/articles/10.1186/s13012-019-0892-4) [strategies to address contextual barriers: diversity](https://implementationscience.biomedcentral.com/articles/10.1186/s13012-019-0892-4) [in recommendations and future directions.](https://implementationscience.biomedcentral.com/articles/10.1186/s13012-019-0892-4) Implementation Science. 2019 Dec;14(1):42. | This paper describes how one might match implementation strategies to determinants. |
|  | |
| **Equity and Implementation Science** | |
| 20. Shelton RC, Chambers DA, Glasgow RE. [An](https://www.frontiersin.org/articles/10.3389/fpubh.2020.00134/full) [extension of RE-AIM to enhance sustainability:](https://www.frontiersin.org/articles/10.3389/fpubh.2020.00134/full) [addressing dynamic context and promoting](https://www.frontiersin.org/articles/10.3389/fpubh.2020.00134/full) [health equity over time. Frontiers in Public](https://www.frontiersin.org/articles/10.3389/fpubh.2020.00134/full) [Health.](https://www.frontiersin.org/articles/10.3389/fpubh.2020.00134/full) 2020;8:134. | This paper proposes an extension to the RE-AIM framework that explicitly attends to both sustainability and health equity. |
| 21. Woodward EN, Matthieu MM, Uchendu US, Rogal S, Kirchner JE. [The health equity](https://implementationscience.biomedcentral.com/articles/10.1186/s13012-019-0861-y) [implementation framework: proposal and](https://implementationscience.biomedcentral.com/articles/10.1186/s13012-019-0861-y) [preliminary study of hepatitis C virus treatment.](https://implementationscience.biomedcentral.com/articles/10.1186/s13012-019-0861-y) [Implementation Science.](https://implementationscience.biomedcentral.com/articles/10.1186/s13012-019-0861-y) 2019 Dec;14(1):1-8. | This is one of the first implementation science frameworks to explicitly prioritize constructs pertaining to health equity. |
| 22. Shelton, R, Adsul, P, Oh, A. [Recommendations](https://pubmed.ncbi.nlm.nih.gov/34045837/) [for addressing structural racism in implementation](https://pubmed.ncbi.nlm.nih.gov/34045837/) [science: A call to the field.](https://pubmed.ncbi.nlm.nih.gov/34045837/) | This paper offers recommendations for the field of IS to include structural racism as a more explicit focus of the work. |
|  | |
| **Grant Writing** | |
| 23. Proctor EK, Powell BJ, Baumann AA, Hamilton AM, Santens RL. [Writing implementation research](https://implementationscience.biomedcentral.com/articles/10.1186/1748-5908-7-96) [grant proposals: ten key ingredients.](https://implementationscience.biomedcentral.com/articles/10.1186/1748-5908-7-96) *Implement Sci*. 2012;12(7). | This paper offers guidance to people writing grant proposals that include or focus on implementation. |

| **Deimplementation** | |
| --- | --- |
| 1. Montini, T, Graham, I. [Entrenched practices](https://implementationscience.biomedcentral.com/articles/10.1186/s13012-015-0211-7) [and other biases: unpacking the historical,](https://implementationscience.biomedcentral.com/articles/10.1186/s13012-015-0211-7) [economic, professional, and social resistance to](https://implementationscience.biomedcentral.com/articles/10.1186/s13012-015-0211-7) [de-implementation.](https://implementationscience.biomedcentral.com/articles/10.1186/s13012-015-0211-7) *Implement Sci.* 2015;10(24). 2. Norton WE, Chambers DA. [Unpacking the](https://implementationscience.biomedcentral.com/articles/10.1186/s13012-019-0960-9) [complexities of de-implementing inappropriate](https://implementationscience.biomedcentral.com/articles/10.1186/s13012-019-0960-9) [health interventions.](https://implementationscience.biomedcentral.com/articles/10.1186/s13012-019-0960-9) Implementation Science. 2020 Dec;15(1):1-7. | De-implementation is not necessarily the opposite of implementation. These papers discuss some of the challenges to changing behavior to discontinue a practice. |
|  | |
| **Quality Improvement and Implementation Science** | |
| 26. Ovretveit J, Mittman B, Rubenstein L, Ganz DA. [Using implementation tools to design and](https://www.ncbi.nlm.nih.gov/pubmed/28958203) [conduct quality improvement projects for faster](https://www.ncbi.nlm.nih.gov/pubmed/28958203) [and more effective improvement.](https://www.ncbi.nlm.nih.gov/pubmed/28958203) *Int J Health Care Qual Assur.* 2017; 30(8):755-768 | This paper explains how implementation science principles can be useful to people doing healthcare improvement work. |
| 27. Koczwara B, Stover AM, Davies L, Davis MM, Fleisher L, Ramanadhan S, Schroeck FR, Zullig LL, Chambers DA, Proctor E. [Harnessing the Synergy](https://ascopubs.org/doi/full/10.1200/JOP.17.00083) [Between Improvement Science and](https://ascopubs.org/doi/full/10.1200/JOP.17.00083) [Implementation Science in Cancer: A Call to](https://ascopubs.org/doi/full/10.1200/JOP.17.00083) [Action. Journal of oncology practice.](https://ascopubs.org/doi/full/10.1200/JOP.17.00083) 2018 Jun;14(6):335. | This paper provides helpful insights on the similarities and differences between QI and IS. |
