# Supplementary figures and images for: Evaluation of a brief virtual implementation science training program: the Penn Implementation Science Institute
Source: Implement Sci Commun. 2023 Nov 6;4:131. doi: 10.1186/s43058-023-00512-5 (PMC10626776; doi:10.1186/s43058-023-00512-5)

**Additional File 4. Pre- and Post-survey Instruments.**

**
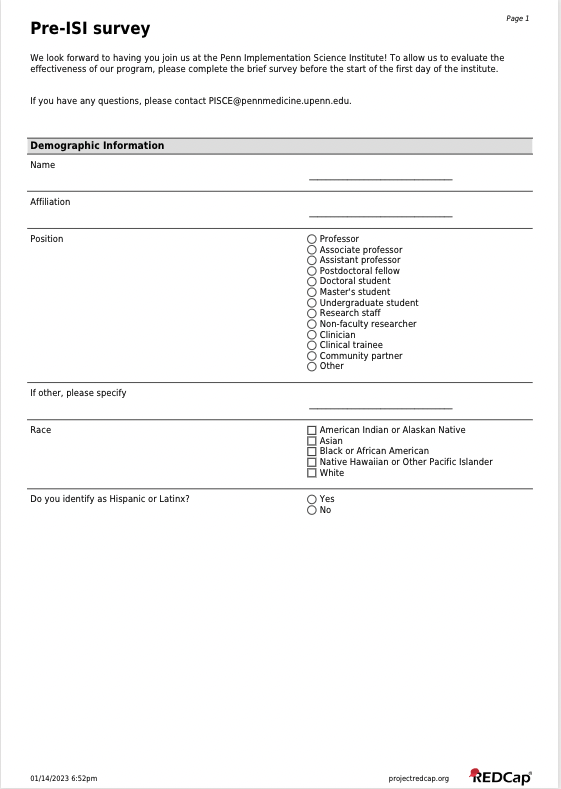
**

**
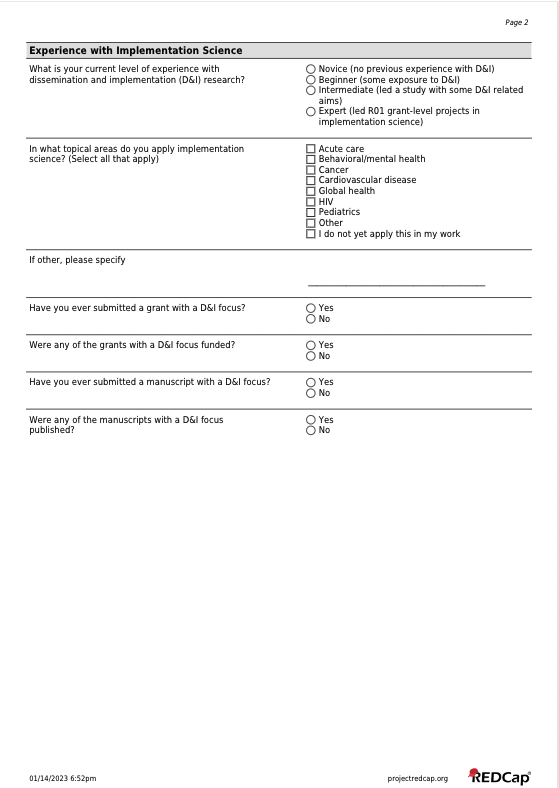

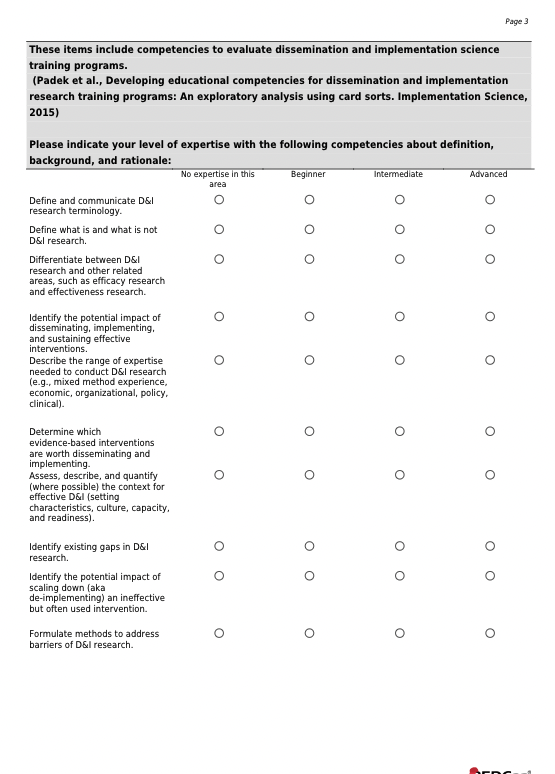

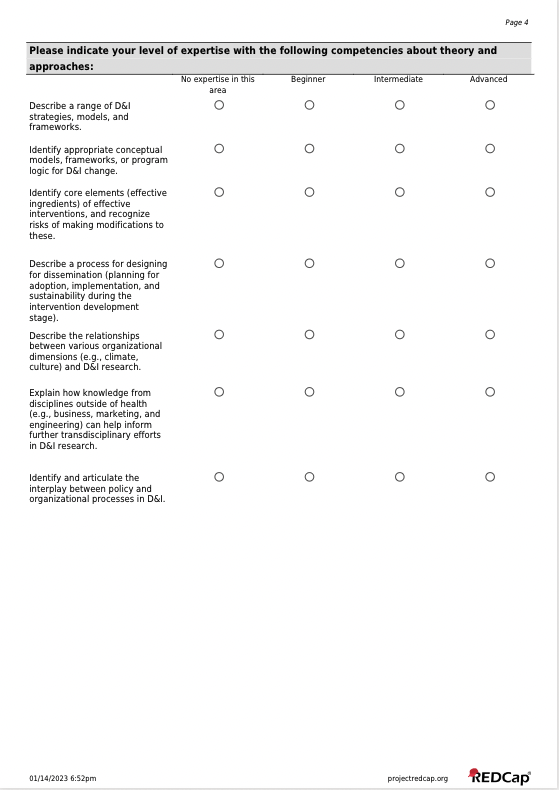

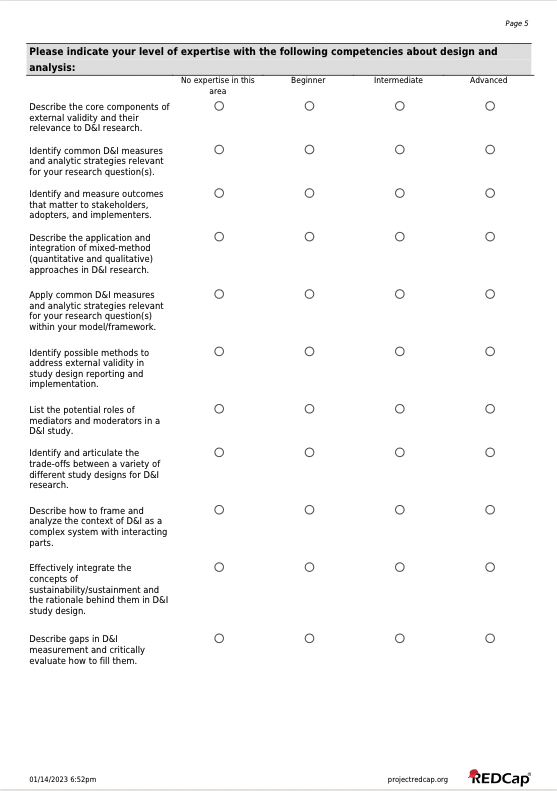

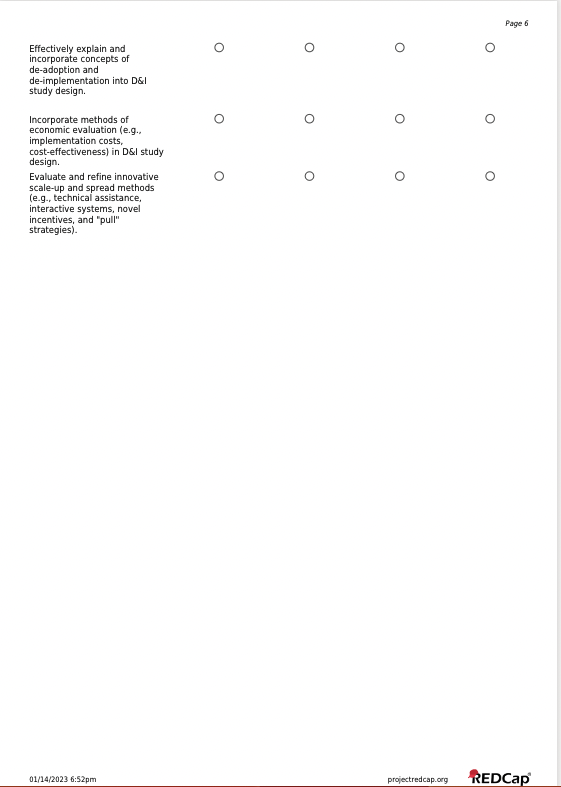

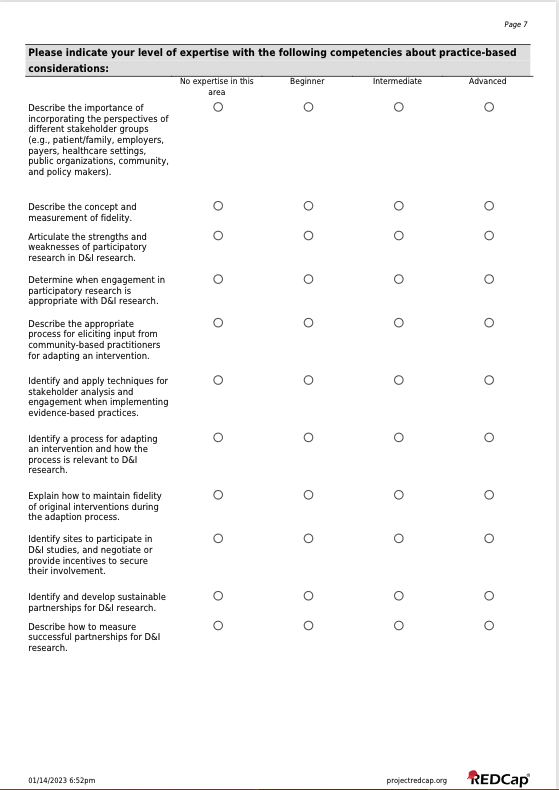

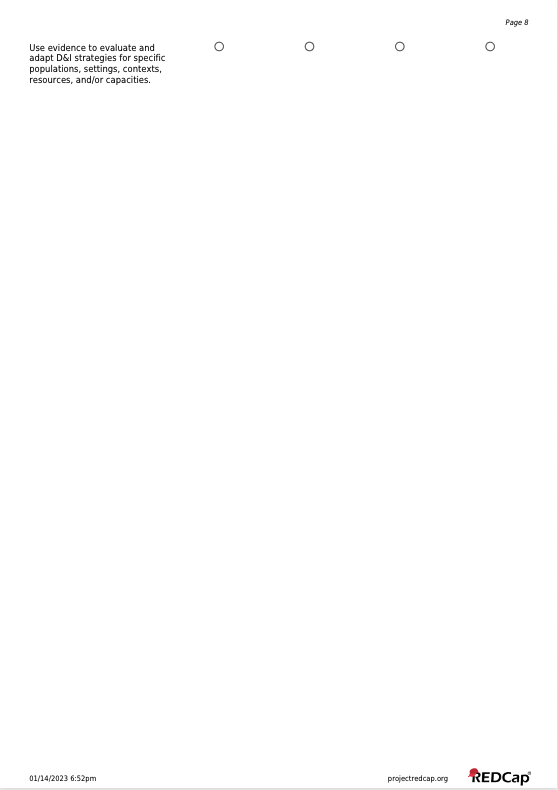

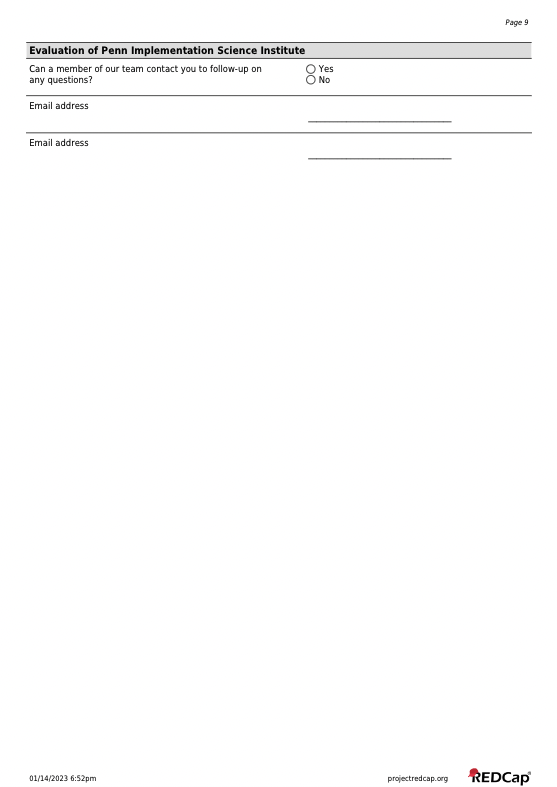

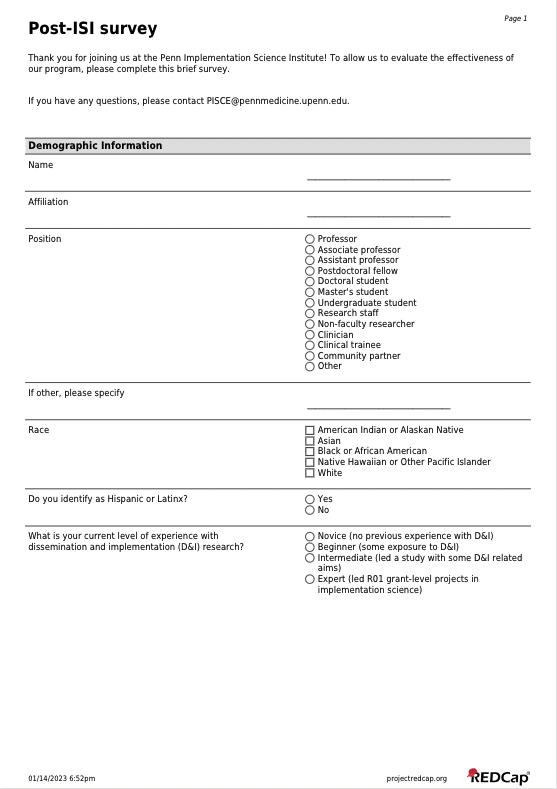

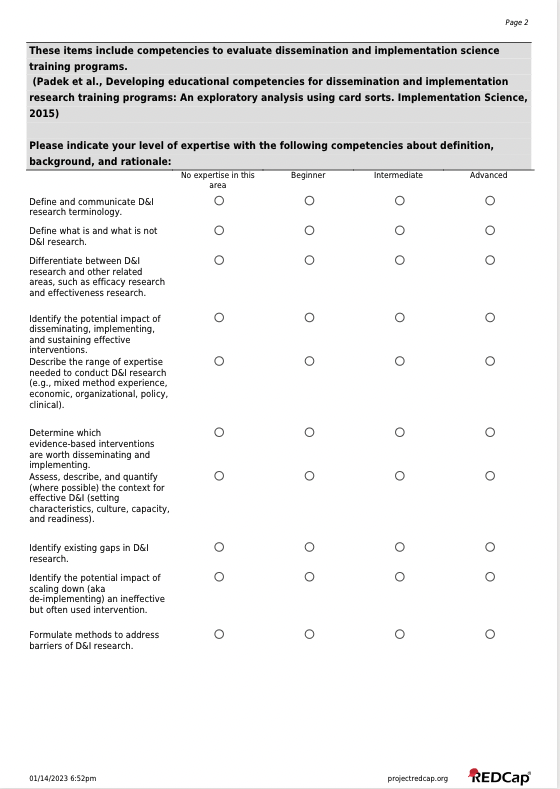

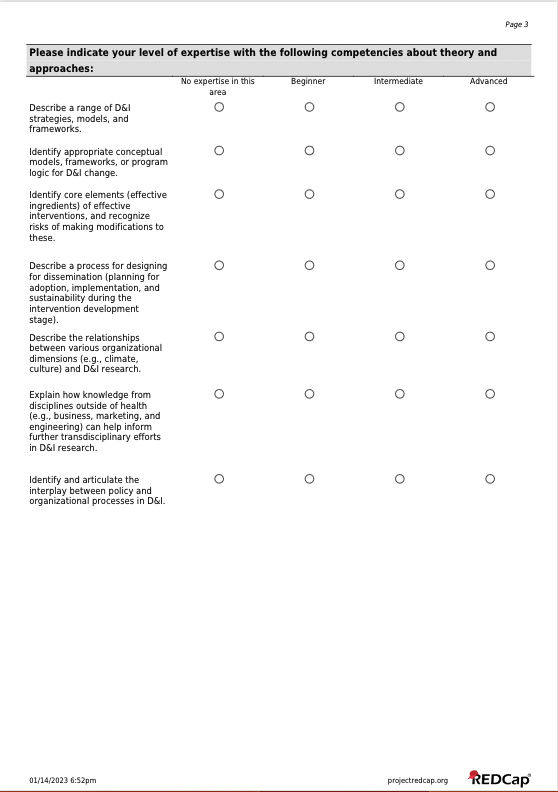

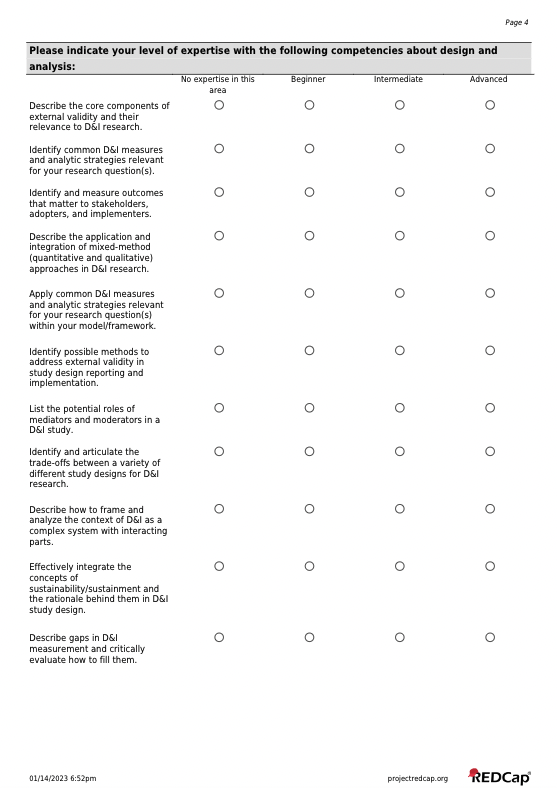

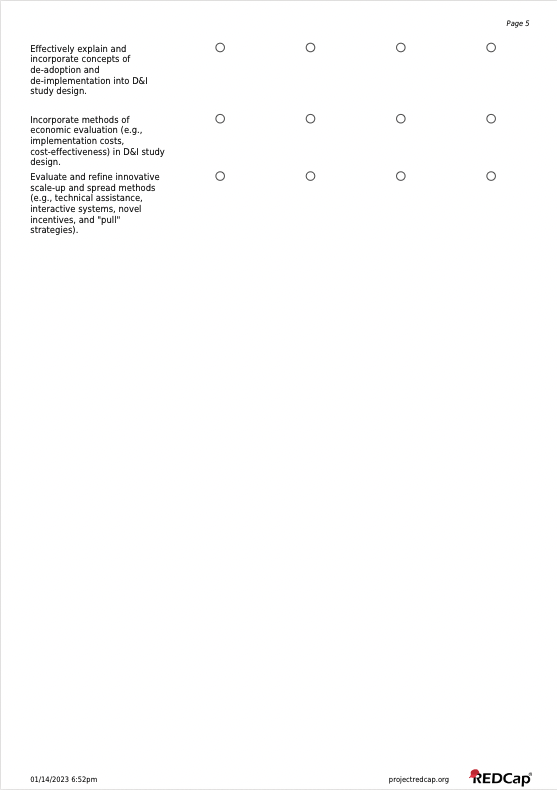

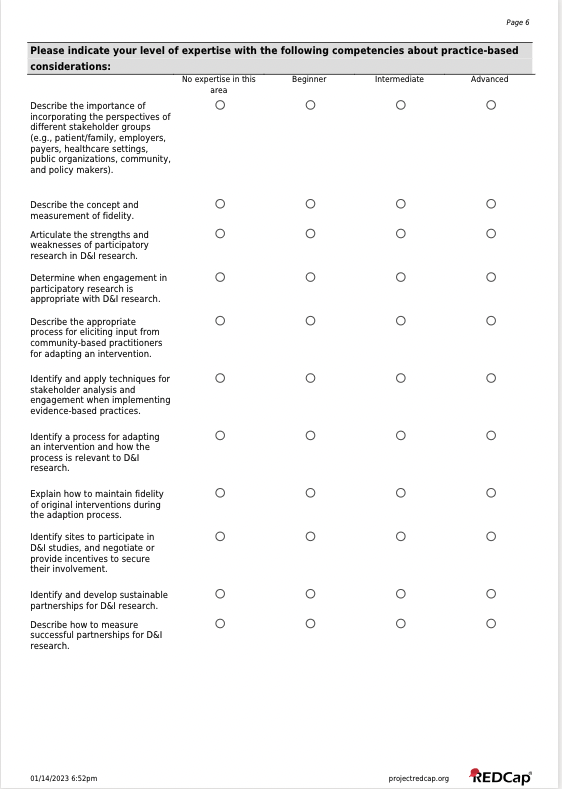

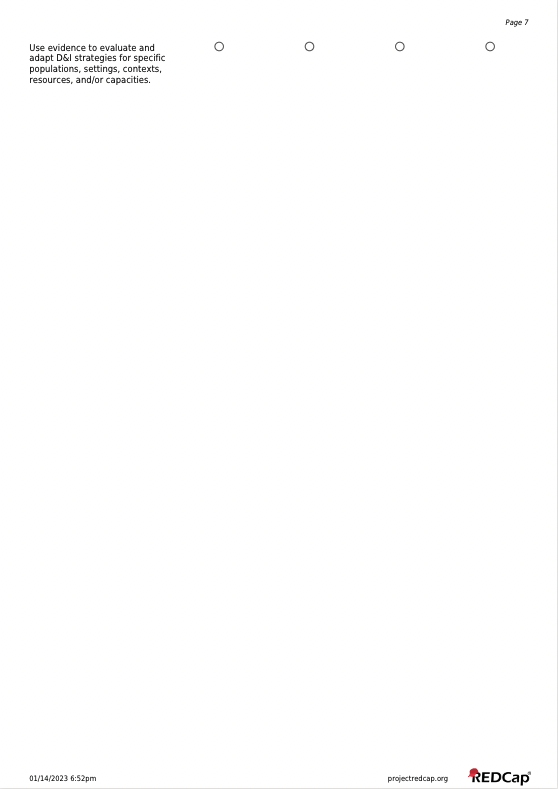

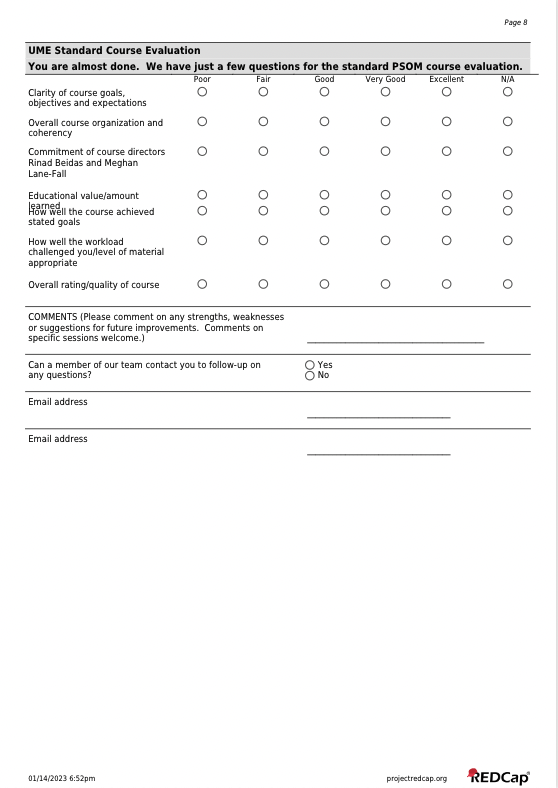
**

Supplement: Supplementary file 4 — Additional file 4. Pre- and Post-survey Instruments. [file 43058_2023_512_MOESM4_ESM.docx]
