# Supplementary material for: Evaluation of a brief virtual implementation science training program: the Penn Implementation Science Institute
Source: Implement Sci Commun. 2023 Nov 6;4:131. doi: 10.1186/s43058-023-00512-5 (PMC10626776; doi:10.1186/s43058-023-00512-5)
Supplement: Supplementary file 5 — Additional file 5. Penn Course Evaluation. [file 43058_2023_512_MOESM5_ESM.docx]

**Additional File 5. Penn Course Evaluation**

| **Standard Course Evaluation Ratings** | | | | | | | |
| --- | --- | --- | --- | --- | --- | --- | --- |
|  | **N (%)** | | | | | |  |
|  | Poor | Fair | Good | Very Good | Excellent | N/A | Mean^*^ |
| Clarity of course goals, objectives, and expectations | 0 | 0 | 1 (2.44) | 15 (36.59) | 25 (60.98) | 0 | 3.59 |
| Overall course organization and coherency | 0 | 0 | 1 (2.44) | 11 (26.83) | 29 (70.73) | 0 | 3.68 |
| Commitment of course directors Rinad Beidas and Meghan Lane-Fall | 0 | 0 | 0 | 1 (2.44) | 40 (97.56) | 0 | 3.98 |
| Educational value/amount learned | 0 | 0 | 2 (4.88) | 9 (21.95) | 30 (73.17) | 0 | 3.68 |
| How well the course achieved stated goals | 0 | 0 | 3 (7.32) | 10 (24.39) | 28 (68.29) | 0 | 3.61 |
| How well the workload challenged you/level of material appropriate | 0 | 1 (2.44) | 4 (9.76) | 7 (17.07) | 28 (68.29) | 1 (2.44) | 3.55 |
| Overall rating/quality of course | 0 | 0 | 0 | 9 (23.08) | 30 (76.92) | 0 | 3.77 |

*For mean calculation, variables were coded as follows: Poor = 0, Fair = 1, Good = 2, Very Good = 3, Excellent = 4; Dropped N/A
