# Supplementary material for: Evaluation of a brief virtual implementation science training program: the Penn Implementation Science Institute
Source: Implement Sci Commun. 2023 Nov 6;4:131. doi: 10.1186/s43058-023-00512-5 (PMC10626776; doi:10.1186/s43058-023-00512-5)
Supplement: Supplementary file 6 — Additional file 6. Individual Implementation Science Competencies by Theme. [file 43058_2023_512_MOESM6_ESM.docx]

**Additional File 6. Individual Implementation Science Competencies by Theme**

| **Competencies on D&I Definition, Background, and Rationale** | | | | | | | | | | | | | | | | | | | | | | | | | | | | | | | | | | | | | | | | | | | | | | | |
| --- | --- | --- | --- | --- | --- | --- | --- | --- | --- | --- | --- | --- | --- | --- | --- | --- | --- | --- | --- | --- | --- | --- | --- | --- | --- | --- | --- | --- | --- | --- | --- | --- | --- | --- | --- | --- | --- | --- | --- | --- | --- | --- | --- | --- | --- | --- | --- |
|  | No expertise in this area | | | | | | | | | | | Beginner | | | | | | | | | | | | Intermediate | | | | | | | | | | Advanced | | | | | | | | |  | | | | |
|  | **N (%)** | | | | | | | | | | | | | | | | | | | | | | | | | | | | | | | | | | | | | | | | | | **Mean**^*^ | | | | |
|  | Pre | | | | | | | | | | Post | Pre | | | | | | | | | | Post | | Pre | | | | | | | | | Post | Pre | | | | | | | Post | | Pre | Post | | | |
| Define and communicate D&I research terminology. | 26 (25.24) | | | | | | | | | | 1 (2.33) | 59 (57.28) | | | | | | | | | | | 17 (39.53) | 16 (15.53) | | | | | | | | | 24 (55.81) | 2 (1.94) | | | | | | | | 1 (2.33) | 0.94 | | | | 1.58 |
| Define what is and what is not D&I research. | 25 (24.51) | | | | | | | | | 1 (2.33) | | 55 (53.92) | | | | | | | | | 17 (39.53) | | | 18 (17.65) | | | | | | | | 24 (55.81) | | 4 (3.92) | | | | | | 1 (2.33) | | | 1.01 | | | 1.58 | |
| Differentiate between D&I research and other related areas, such as efficacy research and effectiveness research. | 28 (27.18) | | | | | | | | 2 (4.65) | | | 53 (51.46) | | | | | | | | 14 (32.56) | | | | 18 (17.48) | | | | | | | 24 (55.81) | | | 4 (3.88) | | | | | 3 (6.98) | | | | 0.98 | | | 1.65 | |
| Identify the potential impact of disseminating, implementing, and sustaining effective interventions. | 21 (20.59) | | | | | | | 0 | | | | 58 (56.86) | | | | | | | 18 (41.86) | | | | | 19 (18.63) | | | | | | 23 (53.49) | | | | 4 (3.92) | | | | 2 (4.65) | | | | | 1.06 | | 1.63 | | |
| Describe the range of expertise needed to conduct D&I research (e.g., mixed method experience, economic, organizational, policy, clinical). | 40 (38.83) | | | | | | 1 (2.33) | | | | | 48 (46.60) | | | | | | 17 (39.53) | | | | | | 15 (14.56) | | | | | 23 (53.49) | | | | | 0 | | | 2 (4.65) | | | | | | 0.76 | | 1.60 | | |
| Determine which evidence-based interventions are worth disseminating and implementing. | 33 (32.04) | | | | | 2 (4.88) | | | | | | 48 (46.60) | | | | | 17 (41.46) | | | | | | | 18 (17.48) | | | | 22 (53.66) | | | | | | 4 (3.88) | | | 0 | | | | | | 0.93 | | 1.49 | | |
| Assess, describe, and quantify (where possible) the context for effective D&I (setting characteristics, culture, capacity, and readiness). | 42 (40.78) | | | | 2 (4.65) | | | | | | | 49 (47.57) | | | | 19 (44.19) | | | | | | | | 11 (10.68) | | | 21 (48.84) | | | | | | | 1 (0.97) | | 1 (2.33) | | | | | | | 0.72 | | 1.49 | | |
| Identify existing gaps in D&I research. | 53 (51.46) | | | 3 (6.98) | | | | | | | | 35 (33.98) | | | 25 (58.14) | | | | | | | | | 15 (14.56) | | 14 (32.56) | | | | | | | | 0 | 1 (2.33) | | | | | | | | 0.63 | | 1.30 | | |
| Identify the potential impact of scaling down (aka de-implementing) an ineffective but often used intervention. | 61 (59.22) | | 3 (7.14) | | | | | | | | | 35 (33.98) | | 24 (57.14) | | | | | | | | | | 7 (6.80) | 15 (35.71) | | | | | | | | | 0 | 0 | | | | | | | | 0.48 | | 1.29 | | |
| Formulate methods to address barriers of D&I research. | 50 (48.54) | 3 (6.98) | | | | | | | | | | 45 (43.69) | 22 (51.16) | | | | | | | | | | | 8 (7.77) | 17 (39.53) | | | | | | | | | 0 | 1 (2.33) | | | | | | | | 0.59 | | 1.37 | | |

*For mean calculation, variables were coded as follows: No expertise in this area = 0, Beginner = 1, Intermediate = 2, Advanced = 3

| **Competencies on D&I Theory and Approaches** | | | | | | | | | | | | | | | | | | | | | | | | | | | | | | | | | | | | |
| --- | --- | --- | --- | --- | --- | --- | --- | --- | --- | --- | --- | --- | --- | --- | --- | --- | --- | --- | --- | --- | --- | --- | --- | --- | --- | --- | --- | --- | --- | --- | --- | --- | --- | --- | --- | --- |
|  | No expertise in this area | | | | | | | | Beginner | | | | | | | | Intermediate | | | | | | | | Advanced | | | | | | | |  | | | |
|  | **N (%)** | | | | | | | | | | | | | | | | | | | | | | | | | | | | | | | | **Mean** | | | |
|  | Pre | | | | | | | Post | Pre | | | | | | | Post | Pre | | | | | | | Post | Pre | | | | | | Post | | Pre | Post | | |
| Describe a range of D&I strategies, models, and frameworks. | 38 (36.89) | | | | | | | 1 (2.33) | 54 (52.43) | | | | | | | 17 (39.53) | 10 (9.71) | | | | | | | 24 (55.81) | 1 (0.97) | | | | | | | 1 (2.33) | 0.75 | | | 1.58 |
| Identify appropriate conceptual models, frameworks, or program logic for D&I change. | 46 (44.66) | | | | | | 2 (4.65) | | 48 (46.60) | | | | | | 19 (44.19) | | 8 (7.77) | | | | | | 21 (48.84) | | 1  (0.97) | | | | | 1 (2.33) | | | 0.65 | | 1.49 | |
| Identify core elements (effective ingredients) of effective interventions, and recognize risks of making modifications to these. | 41 (40.20) | | | | | 3 (6.98) | | | 43 (42.16) | | | | | 22 (51.16) | | | 15 (14.71) | | | | | 18 (41.86) | | | 3 (2.94) | | | | 0 | | | | 0.80 | 1.35 | | |
| Describe a process for designing for dissemination (planning for adoption, implementation, and sustainability during the intervention development stage). | 45 (43.69) | | | | 2 (4.65) | | | | 41 (39.81) | | | | 22 (51.16) | | | | 15 (14.56) | | | | 19 (44.19) | | | | 2 (1.94) | | | 0 | | | | | 0.75 | 1.40 | | |
| Describe the relationships between various organizational dimensions (e.g., climate, culture) and D&I research. | 49 (47.57) | | | 2 (4.65) | | | | | 40 (38.83) | | | 24 (55.81) | | | | | 13 (12.62) | | | 16 (37.21) | | | | | 1  (0.97) | | 1 (2.33) | | | | | | 0.67 | 1.37 | | |
| Explain how knowledge from disciplines outside of health (e.g., business, marketing, and engineering) can help inform further transdisciplinary efforts in D&I research. | 56 (54.37) | | 3 (6.98) | | | | | | 42 (40.78) | | 21 (48.84) | | | | | | 5 (4.85) | | 19 (44.19) | | | | | | 0 | 0 | | | | | | | 0.50 | 1.37 | | |
| Identify and articulate the interplay between policy and organizational processes in D&I. | 56 (54.37) | 3 (6.98) | | | | | | | 42 (40.78) | 25 (58.14) | | | | | | | 5 (4.85) | 15 (34.88) | | | | | | | 0 | 0 | | | | | | | 0.50 | 1.30 | | |

*For mean calculation, variables were coded as follows: No expertise in this area = 0, Beginner = 1, Intermediate = 2, Advanced = 3

| **Competencies on D&I Design and Analysis** | | | | | | | | | | | | | | | | | | | | | | | | | | |
| --- | --- | --- | --- | --- | --- | --- | --- | --- | --- | --- | --- | --- | --- | --- | --- | --- | --- | --- | --- | --- | --- | --- | --- | --- | --- | --- |
|  | No expertise in this area | | Beginner | | Intermediate | | | | | | | | Advanced | | | | | | | | | | | |  | |
|  | **N (%)** | | | | | | | | | | | | | | | | | | | | | | | | **Mean** | |
|  | Pre | Post | Pre | Post | Pre | | | | | | | Post | Pre | | | | | | | | | | | Post | Pre | |
| Describe the core components of external validity and their relevance to D&I research. | 48 (48.48) | 2 (4.76) | 40 (40.40) | 24 (57.14) | 11 (11.11) | | | | | | 16 (38.10) | | 0 | | | | | | | | | | 0 | | | 0.63 |
| Identify common D&I measures and analytic strategies relevant for your research question(s). | 50 (49.50) | 1 (2.38) | 42 (41.58) | 19 (45.24) | 8 (7.92) | | | | | | 21 (50.00) | | 1 (0.99) | | | | | | | | | | 1 (2.38) | | 0.60 | |
| Identify and measure outcomes that matter to stakeholders, adopters, and implementers. | 36 (35.29) | 0 | 44 (43.14) | 20 (48.78) | 18 (17.65) | | | | | 20 (48.78) | | | 4 (3.92) | | | | | | | | | | 1 (2.44) | | 0.90 | |
| Describe the application and integration of mixed-method (quantitative and qualitative) approaches in D&I research. | 40 (39.60) | 3 (7.14) | 48 (47.52) | 17 (40.48) | 10 (9.90) | | | | 20 (47.62) | | | | 3 (2.97) | | | | | | | | | 2 (4.76) | | | 0.76 | |
| Apply common D&I measures and analytic strategies relevant for your research question(s) within your model/framework. | 51 (50.00) | 3 (7.14) | 40 (39.22) | 24 (57.14) | 10 (9.80) | 14 (33.33) | | | | | | | 1 (0.98) | | | | | | | | 1 (2.38) | | | | 0.62 | |
| Identify possible methods to address external validity in study design reporting and implementation. | 56 (54.90) | 4 (9.52) | 37 (36.27) | 25 (59.52) | 8 (7.84) | | | 12 (28.57) | | | | | 1 (0.98) | | | | | | | 1 (2.38) | | | | | 0.55 | |
| List the potential roles of mediators and moderators in a D&I study. | 64 (62.75) | 4 (9.52) | 32 (31.37) | 25 (59.52) | 5 (4.90) | | 13 (30.95) | | | | | | 1 (0.98) | | | | | | 0 | | | | | | 0.44 | |
| Identify and articulate the trade-offs between a variety of different study designs for D&I research. | 61 (60.40) | 3 (7.14) | 32 (31.68) | 26 (61.90) | 7 (6.93) | | 13 (30.95) | | | | | | 1 (0.99) | | | | | 0 | | | | | | | 0.49 | |
| Describe how to frame and analyze the context of D&I as a complex system with interacting parts. | 62 (61.39) | 3 (7.50) | 34 (33.66) | 20 (50.00) | 4 (3.96) | | 17 (42.50) | | | | | | 1 (0.99) | | | | 0 | | | | | | | | 0.45 | |
| Effectively integrate the concepts of sustainability/sustainment and the rationale behind them in D&I study design. | 58 (58.00) | 5 (11.90) | 33 (33.00) | 22 (52.38) | 8 (8.00) | | 15 (35.71) | | | | | | 1 (1.00) | | | 0 | | | | | | | | | 0.52 | |
| Describe gaps in D&I measurement and critically evaluate how to fill them. | 69 (67.65) | 5 (11.90) | 29 (28.43) | 23 (54.76) | 3 (2.94) | | 14 (33.33) | | | | | | 1 (0.98) | | 0 | | | | | | | | | | 0.37 | |
| Effectively explain and incorporate concepts of de-adoption and de-implementation into D&I study design. | 72 (70.59) | 3 (7.32) | 27 (26.47) | 26 (63.41) | 3 (2.94) | | 12 (29.27) | | | | | | 0 | 0 | | | | | | | | | | | 0.32 | |
| Incorporate methods of economic evaluation (e.g., implementation costs, cost-effectiveness) in D&I study design. | 67 (65.69) | 5 (11.90) | 32 (31.37) | 29 (69.05) | 3 (2.94) | | 8 (19.05) | | | | | | 0 | 0 | | | | | | | | | | | 0.37 | |
| Evaluate and refine innovative scale-up and spread methods (e.g., technical assistance, interactive systems, novel incentives, and 'pull' strategies). | 67 (65.69) | 7 (16.67) | 32 (31.37) | 24 (57.14) | 3 (2.94) | | 11 (26.19) | | | | | | 0 | 0 | | | | | | | | | | | 0.37 | |

*For mean calculation, variables were coded as follows: No expertise in this area = 0, Beginner = 1, Intermediate = 2, Advanced = 3

| **Competencies on D&I Practice-based Considerations** | | | | | | | | | | | |
| --- | --- | --- | --- | --- | --- | --- | --- | --- | --- | --- | --- |
|  | No expertise in this area | | | Beginner | | Intermediate | | Advanced | |  | |
|  | **N (%)** | | | | | | | | | **Mean** | |
|  | Pre | Post | | Pre | Post | Pre | Post | Pre | Post | Pre | Post |
| Describe the importance of incorporating the perspectives of different stakeholder groups (e.g., patient/family, employers, payers, healthcare settings, public organizations, community, and policy makers). | 21 (20.59) | 1 (2.44) | | 45 (44.12) | 12 (29.27) | 31 (30.39) | 22 (53.66) | 5 (4.90) | 6 (14.63) | 1.20 | 1.80 |
| Describe the concept and measurement of fidelity. | 33 (32.35) | | 1 (2.44) | 43 (42.16) | 13 (31.71) | 22 (21.57) | 24 (58.54) | 4 (3.92) | 3 (7.32) | 0.97 | 1.71 |
| Articulate the strengths and weaknesses of participatory research in D&I research. | 53 (51.96) | | 1 (2.44) | 34 (33.33) | 21 (51.22) | 14 (13.73) | 16 (39.02) | 1 (0.98) | 3 (7.32) | 0.64 | 1.51 |
| Determine when engagement in participatory research is appropriate with D&I research. | 54 (52.94) | | 2 (4.88) | 37 (36.27) | 22 (53.66) | 10 (9.80) | 14 (34.15) | 1 (0.98) | 3 (7.32) | 0.59 | 1.44 |
| Describe the appropriate process for eliciting input from community-based practitioners for adapting an intervention. | 50 (49.50) | | 3 (7.32) | 36 (35.64) | 16 (39.02) | 14 (13.86) | 21 (51.22) | 1 (0.99) | 1 (2.44) | 0.66 | 1.49 |
| Identify and apply techniques for stakeholder analysis and engagement when implementing evidence-based practices. | 48 (47.06) | | 1 (2.44) | 37 (36.27) | 23 (56.10) | 15 (14.71) | 16 (39.02) | 2 (1.96) | 1 (2.44) | 0.72 | 1.41 |
| Identify a process for adapting an intervention and how the process is relevant to D&I research. | 47 (46.53) | | 2 (4.88) | 40 (39.60) | 20 (48.78) | 13 (12.87) | 19 (46.34) | 1 (0.99) | 0 | 0.68 | 1.41 |
| Explain how to maintain fidelity of original interventions during the adaption process. | 52 (50.98) | | 3 (7.32) | 37 (36.27) | 24 (58.54) | 12 (11.76) | 14 (34.15) | 1 (0.98) | 0 | 0.63 | 1.27 |
| Identify sites to participate in D&I studies, and negotiate or provide incentives to secure their involvement. | 54 (52.94) | | 3 (7.32) | 38 (37.25) | 23 (56.10) | 9 (8.82) | 15 (36.59) | 1 (0.98) | 0 | 0.58 | 1.29 |
| Identify and develop sustainable partnerships for D&I research. | 55 (53.92) | | 3 (7.32) | 36 (35.29) | 22 (53.66) | 10 (9.80) | 16 (39.02) | 1 (0.98) | 0 | 0.58 | 1.32 |
| Describe how to measure successful partnerships for D&I research. | 66 (65.35) | | 4 (9.76) | 32 (31.68) | 25 (60.98) | 2 (1.98) | 12 (29.27) | 1 (0.99) | 0 | 0.37 | 1.20 |
| Use evidence to evaluate and adapt D&I strategies for specific populations, settings, contexts, resources, and/or capacities. | 57 (55.88) | | 3 (7.32) | 34 (33.33) | 20 (48.78) | 10 (9.80) | 18 (43.90) | 1 (0.98) | 0 | 0.56 | 1.37 |

*For mean calculation, variables were coded as follows: No expertise in this area = 0, Beginner = 1, Intermediate = 2, Advanced = 3
